# Compound maximal motor unit response is modulated by contraction intensity, but not contraction type in tibialis anterior

**DOI:** 10.14814/phy2.14201

**Published:** 2019-09-08

**Authors:** Jamie Tallent, Stuart Goodall, Dawson J. Kidgell, Rade Durbaba, Glyn Howatson

**Affiliations:** ^1^ School of Sport Health and Applied Science St Mary's University Twickenham United Kingdom; ^2^ Faculty of Health and Life Sciences Northumbria University Newcastle‐upon‐Tyne United Kingdom; ^3^ Department of Physiotherapy, School of Primary Health Care, Faculty of Medicine, Nursing and Health Sciences Monash University Melbourne Australia; ^4^ Water Research Group, School of Biological Sciences North West University Potchefstroom South Africa

**Keywords:** Eccentric contractions, electromyography, *M*_MAX_, peripheral nerve stimulation

## Abstract

Determining a single compound maximal motor response (*M*_MAX_) or an average superimposed *M*_MAX_ response (*M*_SUP_) are commonly used reference values in experiments eliciting raw electromyographic, motor evoked potentials, H‐reflexes, and V‐waves. However, existing literature is limited in detailing the most appropriate method to normalize these electrophysiological measures. Due to the accessibility of assessment from a cortical and spinal perspective, the tibialis anterior is increasingly used in literature and hence investigated in this study. The aims of the present study were to examine the differences and level of agreement in *M*_MAX_/*M*_SUP_ under different muscle actions and contraction intensities. Following a familiarization session, 22 males visited the laboratory on a single occasion. *M*_MAX_ was recorded under 10% isometric and 25% and 100% shortening and lengthening maximal voluntary contractions (MVC) at an angular velocity of 15° sec^−^^1^. *M*_SUP_ was also recorded during 100% shortening and lengthening with an average of five responses recorded. There were no differences in *M*_MAX_ or *M*_SUP_ between contraction types. All variables showed large, positive correlations (*P* < 0.001, *r* ^2^ ≥ 0.64). *M*_MAX_ amplitude was larger (*P* < 0.001) at 100% shortening and lengthening intensity compared to *M*_MAX_ amplitude at 10% isometric and 25% lengthening MVC. Bland‐Altman plots revealed a bias toward higher *M*_MAX_ at the higher contraction intensities. Despite *M*_SUP_ being significantly smaller than *M*_MAX_ (*P* < 0.001) at 100% MVC, *M*_SUP_ showed a large positive correlation (*P* < 0.001, *r* ^2^ ≥ 0.64) with all variables. It is our recommendation that *M*_MAX_ should be recorded at specific contraction intensity but not necessarily a specific contraction type.

## Introduction

Electromyographic (EMG) signals are affected by numerous factors such as preparation of the skin, electrode placement, fiber type and orientation (De Luca 1997). It is therefore critical that the EMG signal is normalized to a reference value so that data can be interpreted meaningfully. Applying supramaximal electrical stimulation to a peripheral nerve causes synchronous activation of the muscle fibers and is known as the maximal motor unit response (*M*_MAX_; Lee and Carroll 2005). Investigations using peripherally evoked measures such as the Hoffman‐reflex (H‐reflex) and V‐wave, along with cortically evoked measures such as motor evoked potentials (MEP), lateral spread MEP, and cervicomedullary MEP commonly use *M*_MAX_ as a reference value (Aagaard et al. 2002; Yamashita et al. 2002; Kidgell and Pearce 2010; Tallent et al. 2012a). These spinal and corticospinal measures have been investigated under a variety of conditions such as changing muscle lengths, at rest and during submaximal and maximal contractions (Arányi et al. 1998; Goodall et al. 2009; Howatson et al. 2011; Tallent et al. 2012a). Understanding how *M*_MAX_ is modulated in different muscles, contraction intensities and types is vital in ensuring that EMG is presented in the most appropriate manner.

The *M*_MAX_ amplitude has been shown to increase with increasing contraction intensity in the tibialis anterior (TA: Nagata and Christianson 1995; Frigon et al. 2007) and soleus (Frigon et al. 2007), but remain unchanged in quadriceps muscles (Linnamo et al. 2001a) and in the flexor carpi radialis (Lee and Carroll 2005), or even decreases in the quadriceps (Linnamo et al. 2001b). In addition, *M*_MAX_ has been shown to increase (Gerilovsky et al. 1977; Gerilovsky et al. 1989; Frigon et al. 2007) and decrease when recorded at longer muscle lengths (Marsh et al. 1981; Kim et al. 2005; Lee and Carroll 2005). Furthermore there are conflicting findings in TA with regards to how *M*_MAX_ alters with changing length (Marsh et al. 1981; Frigon et al. 2007). Higher contraction intensities will cause the muscle to shorten and the tendon to become more compliant (Griffiths, 1991). A reduction in muscle length has been shown to cause an increase in synchronization and consequently an increase in *M*_MAX_ (Kim et al. 2005). Alternatively, phase cancellation of EMG will increase with increasing contraction intensity and may mute the response in the muscle (Keenan et al. 2006; Farina et al. 2008). Therefore, understanding *M*_MAX_ response at differing contraction intensities is essential from a clinical and research perspective.

Changes in muscle length might also influence *M*_MAX_ values during shortening and lengthening contractions. It has been recommended (Zehr, 2002) that *M*_MAX_ is expressed relative to the specific muscle action (i.e., shortening or lengthening muscle actions). However, evidence has shown no difference between *M*_MAX_ amplitude when recorded during shortening and lengthening actions (Linnamo et al. 2001b; Duclay and Martin 2005). Ensuring the reference values are recorded to a standardized muscle length appears essential in the interpretation of EMG signals.

V‐wave reflects the efferent neural output during voluntary muscle activation (Aagaard et al. 2002). In the literature, V‐wave is expressed relative to a mean M‐wave (*M*_SUP_) during a number of maximal contractions, (Aagaard et al. 2002; Duclay and Martin 2005; Gondin et al. 2006) or maximal peak‐to‐peak *M*_MAX_ amplitude from the same number of responses (Tallent et al. 2012b, 2013). Due to the increased potential for phase cancellation at higher contraction intensities it is unclear how these different reference values (*M*_MAX_ or *M*_SUP_) affect the outcome and interpretation of the V‐wave. Investigating how *M*_MAX_ is modulated under different muscle actions, and at varying contraction intensities might provide helpful methodological evidence for the use of *M*_MAX_ in experimental paradigms where neurophysiological parameters require normalization.

Therefore, the aim of this study was to investigate changes in *M*_MAX_ under a variety of contraction modes and intensities and examine *M*_SUP_ during maximal shortening and lengthening contractions in the TA. The results from this study will provide guidance for researchers in the use of *M*_MAX_ as a reference value.

## Methods

### Participants

Based on previous work (Kim et al. 2005) examining greater *M*_MAX_ amplitudes during higher contraction intensities (12%; Cohen's *d* = 0.45), a total of 22 participants were recruited for the study to achieve a statistical power of 0.8 with an alpha level of 0.05. Following institutional (Northumbria University) ethical approval, 22 males (mean ± SD, age 23 ± 3 years, stature 178.0 ± 7.0 cm, mass 83.1 ± 9.3 kg) volunteered to participate. After being fully briefed on the experimental protocol and screened for contraindications to the procedures, volunteers provided written informed consent.

### General procedure

Two identical trials were completed, on two consecutive days at the same time of day, with the first trial used to familiarize the participants with the procedures as based on previous recommendations by our laboratory (Tallent et al. 2012a). All contractions were performed on an isokinetic dynamometer (Cybex Norm, Cybex International, NY) that was set up for ankle dorsiflexion of the dominant limb. Footedness was assessed using the questionnaire from Hebbal and Mysorekar (2006). The foot was strapped into an ankle adaptor and the knee was secured into a thigh stabilizer to prevent any extraneous movements. The hip, knee, and ankle were set at joint angles of 90, 120, and 90°, respectively, according to the manufacturer's instructions. Shortening and lengthening contractions consisted of participants moving through a range of 30° (±15° from the ankle at 90°) at an angular velocity of 15°·sec^−1^. Shortening and lengthening contractions began at an ankle angle of 105° and 75° respectively. For shortening muscle actions, participants were instructed to assist the movement of the foot adaptor, and for lengthening the actions required participants to resist movement of the foot adaptor. All responses (torque and EMG) were recorded as the ankle joint passed through anatomical zero (90°). To ensure torque and EMG were recorded at the correct angle, a trigger was set to automatically sweep as the ankle passed 90°. Once secured in the isokinetic dynamometer, participants initially performed shortening, lengthening and isometric MVCs. The highest torque in each muscle action (shortening, lengthening, and isometric) from three trials was recorded as the contraction‐specific MVC.

The *M*_MAX_ was recorded at 10% of isometric, 25% and 100% shortening and lengthening MVC. A 10% isometric contraction is often used to stabilize the H‐reflex in the TA (Griffin & Cafarelli, 2007; Tallent et al. 2012a), and consequently this was considered the resting *M*_MAX_ value. The simulation intensity for eliciting *M*_MAX_ was set at 150% above a plateau in peak‐to‐peak *M*_MAX_ amplitude. This was recorded through an increasing stimulation intensity at 10% isometric MVC and verified during 25% shortening and lengthening contractions. Establishing *M*_MAX_ took around 64 gradually increasing intensity pulses at 10% isometric MVC. *M*_SUP_ was calculated from the average of 5 traces at 100% shortening and lengthening MVC, whilst *M*_MAX_ during a maximal contraction was recorded as the greatest peak‐to‐peak amplitude of the 5 contractions. The order of contraction intensity (10%, 25%, 100%) and type (shortening and lengthening) was randomized.

### Percutaneous nerve stimulation

Searching for optimal site of stimulation began below the head of the fibula, over the peroneal nerve. A 1 msec electrical stimulation was administrated using a 40 mm diameter cathode/anode arrangement (Digitimer DS7AH, Welwyn Garden City, Hertfordshire, UK). Once the optimal site was located, the sight was marked with semi‐permanent ink. The cathode/anode was strapped to the participants’ leg for the entirety of the experiment.

### EMG

Bipolar surface EMG was recorded over the TA using electrodes (22 mm diameter, model; Kendall, Tyco Healthcare Group, Mansfield, MA) spaced 2 cm apart. The reference electrode was placed over the medial malleolus, whilst the TA electrodes were placed at one‐third distance of the line between the tip of the fibula and the tip of the medial malleolus (Hermens et al. 2000). All sites were shaved, abraded, and then wiped clean with an alcohol swab prior to electrode placement. The EMG was amplified (×1000), band pass filtered (10–1000 Hz), and sampled at 5 kHz (CED Power 1401, Cambridge Electronic Design, Cambridge, UK). M‐waves were recorded during a 500 msec window, starting 50 msec before anatomical zero. Once *M*_MAX_ stimulator was established, all further analyses were performed off‐line.

### Torque

To ensure that participants reached the required target torque level, real time feedback was provided on a computer monitor positioned 1 m away. Live feedback was displayed on the monitor of the dynamometer to provide feedback on target forces to achieve during each condition. The torque signal was sampled at 5 kHz, extracted from the dynamometer and synchronized with the EMG signal and analysed off line (Signal v3.0, Cambridge Electronics, Cambridge, UK).

### Statistics

A one‐way ANOVA was used to detect differences between *M*_MAX_ at 10% isometric MVC, 25%, 100% and *M*_SUP_ at 100% shortening and lengthening MVC. Where necessary, LSD post‐hoc analysis was used to make pairwise comparisons with 95% CI (SPSS, v20.0, Chicago, IL). Coefficient of determination and the limits of agreement (Bland and Altman 1986) with 95% CI were also calculated between the variables (GraphPad Software Inc, La Jolla, CA). Correlation coefficients were determined as 0.0–0.1 = trivial, 0.10–0.3, small, 0.3–0.5 = moderate, 0.5–0.7 = large, and 0.7–0.9 = very large (Hopkins, 2009). Effect sizes (*η* ^2^) were defined as: 0.2 trivial, 0.21–0.6 = small, 0.61–1.2 = moderate, 1.21–1.99 = large; >2.0 = very large.

## Results

Isometric contractions were conducted at an average of 8.28 ± 3.21% (target = 10%) of isometric MVC, shortening at 26.1 ± 3.66% (target = 25%), 95.6 ± 11.8% (target = 100%) of shortening MVC and lengthening at 27.1 ± 4.12% (target = 25%), 96.2 ± 9.97% (target = 100%) of lengthening MVC. There was no significant difference (*P* > 0.05) between lengthening and shortening contraction intensities, showing that contractions were conducted at the same relative intensity.

**Figure 1:**
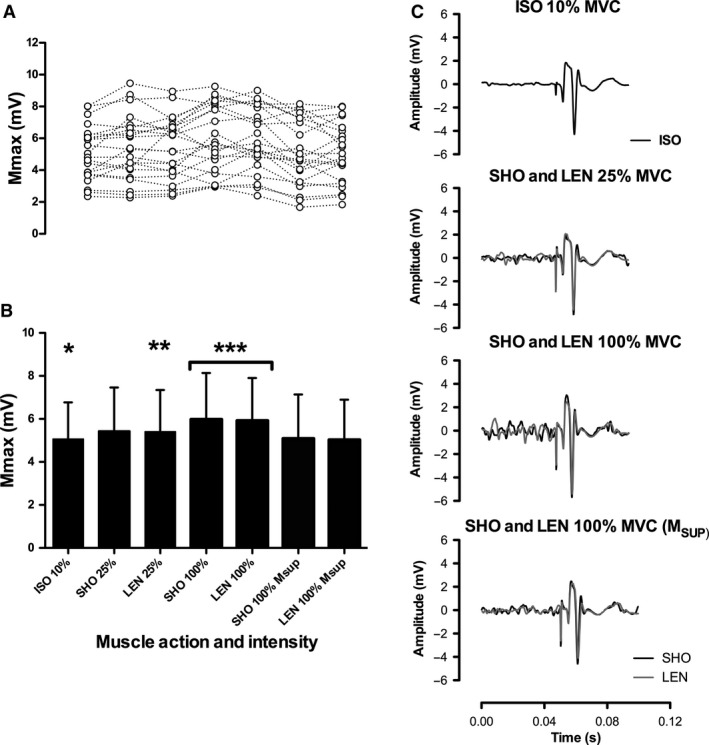
Clear dots represent individual responses at different *M*_MAX_ contraction intensities and contraction types (A). Bars represent mean *M*_MAX_ and *M*_SUP_ responses (mean ± SD) (B). Representative trace from a single participant of *M*_MAX_ recorded at 10% ISO, SHO and LEN 25% and 100% MVC, SHO and LEN *M*_SUP_ (C). ISO, Isometric, SHO, Shortening, LEN, lengthening; *denotes significantly (*P* < 0.05) different from 25% and 100%, SHO and LEN MVC *M*_MAX_; **denotes significantly different from 100% SHO and LEN *M*_MAX_; ***denotes significantly different from SHO and LEN *M*_SUP_.

Figure 1 shows individual and average *M*_MAX_/*M*_SUP_ amplitudes during varying isometric, shortening, and lengthening contractions intensities and a reprehensive trace. The ANOVA revealed there were significant differences in *M*_MAX_ amplitude between conditions (*F*_(6)_ = 6.96: *P* < 0.001; *η* ^2^ = 0.25). Post Hoc analysis showed 10% isometric M_MAX_ MVC was significantly lower than 25% shortening *M*_MAX_ (*P* = 0.03; 95% CI; −0.03 to −0.69 mV), 25% lengthening *M*_MAX_ (*P* = 0.03; 95% CI; −0.05 to −0.64 mV), 100% shortening *M*_MAX_ (*P* < 0.01; 95% CI; −0.40 to −1.32 mV), 100% lengthening *M*_MAX_ (*P* < 0.01; 95% CI; −0.37 to −1.32 mV). *M*_MAX_ was significantly higher at 100% shortening (*P* = 0.02; 95% CI; 0.11 to 1.03 mV) and 100% lengthening (*P* = 0.02; 95% CI; 0.08–1.03) compared to 25% lengthening *M*_MAX_.

**Figure 2:**
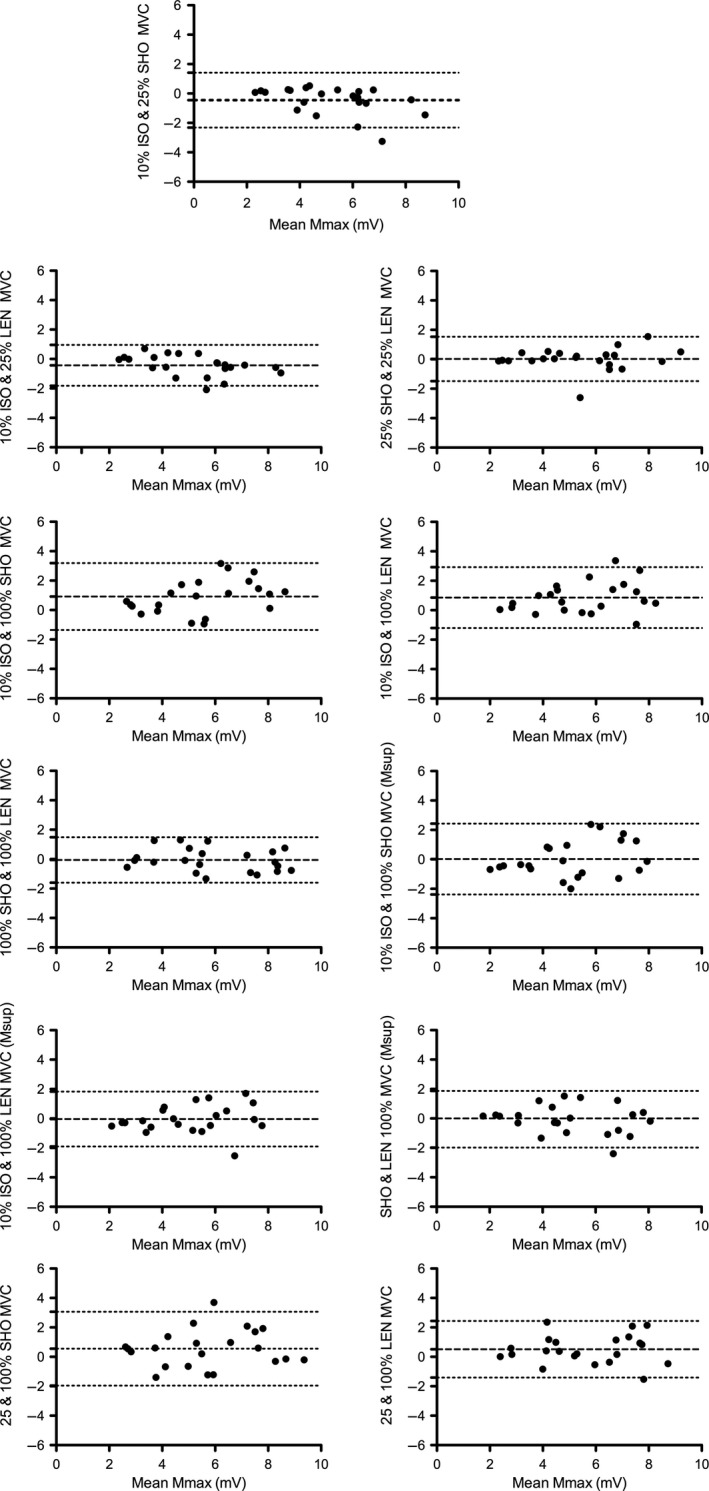
Bland‐Altman plots for *M*_MAX_ and *M*_SUP_ (mV) across varying contraction intensities and type. Dashed line indicated change in mean with 95% confidence intervals. Dots represent individual responses.

All *M*_MAX_ amplitudes were significantly (*P* < 0.001) correlated across intensities (*r* ^2^, ≥0.64). The highest correlations were between contraction types at the same intensity with *M*_MAX_ (100% MVC *r* ^2^ = 0.87; 25% MVC *r* ^2^ = 0.86). Bland‐Altman plots showed a bias toward higher *M*_MAX_ values at higher contraction intensities (Fig. 2). There was no bias between shortening and lengthening contractions. Similarly, isometric *M*_MAX_ and *M*_SUP_ showed no bias.

## Discussion

It has been reported that EMG should be normalized to *M*_MAX_ under the same muscle action and contraction intensity (Zehr, 2002; Duclay and Martin 2005). This study offers further insight into the influence that contraction conditions may affect the amplitude of *M*_MAX_ amplitude. Specifically, the main findings were, (1) there was no difference between *M*_MAX_ amplitudes when recorded at like‐intensities during shortening and lengthening contractions; (2) *M*_MAX_ was influenced by intensity of the contraction, with an increase and systematic bias to an increase *M*_MAX_ during higher intensity contractions; and (3) *M*_MAX_ at 100% MVC was greater compared to *M*_SUP_ at 100% MVC. However, *M*_SUP_ was not different to *M*_MAX_ at 10% MVC, showing little systematic bias and was strongly correlated (*r* ^2^ ≥ 0.64).

It has been recommended that when using *M*_MAX_ as a reference value, it should be recorded under the same contraction intensity as the variable being investigated (Zehr, 2002). The results in this study indicated that with increased contraction intensity the peak‐to‐peak *M*_MAX_ amplitude increased. Previous work has shown similar results in contraction intensities ranging from 40 to 80% isometric MVC in TA (Nagata and Christianson 1995) and 10–30% isometric MVC in TA and the soleus (Frigon et al. 2007). Although this effect is reported previously and supported by the current study, the exact mechanisms for this remain unclear. Frigon et al. (2007) suggested that *M*_MAX_ increased at higher contraction intensities because the muscle length has been shown to be up to 28% shorter at the same joint angle (Griffiths, 1991), and thus, could improve the synchronization of the action potential. However, contrary with our findings, other authors have reported no change (Linnamo et al. 2001a; Lee and Carroll 2005) or even a decrease (Linnamo et al. 2001b) in *M*_MAX_ with increasing contraction intensities. The high degree of variability between subjects might explain why the literature offers little consistency (Lee and Carroll 2005). In addition, phase cancellation has been shown to reduce the EMG response at the muscle during higher contraction intensities (Keenan et al. 2006; Farina et al. 2008). If the responses in EMG are muted at higher contraction intensities then the lack of change in *M*_MAX_ amplitude appears to be associated with the limitations in surface EMG recording (Farina et al. 2014).

Our results support previous findings that showed no difference in *M*_MAX_ under shortening and lengthening contractions (Linnamo et al. 2001b; Duclay and Martin 2005) when measured at the same joint angle. Supramaximal stimulation of the peripheral nerve at shorter muscle lengths improves the synchronization of the action potential (Kim et al. 2005). With an enhanced synchronization of the action potential there is an increase in *M*_MAX_ (Kim et al. 2005). However, the varying pennation angle of muscles might explain why not all studies have found increases in *M*_MAX_ at shorter muscle lengths (Gerilovsky et al. 1977; Gerilovsky et al. 1989; Frigon et al. 2007). Furthermore, it is expected that an increase in *M*_MAX_ at shorter muscle lengths should be associated with decreased duration of *M*_MAX_, although this is not consistently observed (Frigon et al. 2007). In our study, *M*_MAX_ was recorded during shortening and lengthening muscle contractions and importantly, electrical stimulation was delivered at the same joint angle, with the assumption that the muscle was at the same length. Furthermore, current data also showed a strong positive correlation, and a good level of agreement, between *M*_MAX_ during shortening and lengthening muscle actions. Thus, it appears that EMG signals do not necessarily need to be expressed relative to a contraction specific *M*_MAX_, rather, the joint angle should be consistent (Nagata and Christianson 1995; Kim et al. 2005; Frigon et al. 2007). A high level of agreement and a strong correlation was found between shortening and lengthening muscle actions, despite lengthening muscle actions generating a higher level of absolute torque. The differences in *M*_MAX_ at an ‘absolute’ torque might explain why there is a small discrepancy between shortening and lengthening *M*_MAX_ at the same relative intensity.

Unlike MEP's, H‐reflex, and EMG signals, V‐wave is expressed relative to an *M*_SUP_ (Aagaard et al. 2002; Duclay and Martin 2005; Gondin et al. 2006) or *M*_MAX_ (Tallent et al. 2012b; 2013). In this study, there was no difference in *M*_MAX_ at a low intensity contraction (≤25%) and *M*_SUP_. This would suggest that EMG/V‐waves recorded during an MVC could be expressed relative to a low intensity *M*_MAX_ contraction. There was also good level of agreement between *M*_SUP_ and *M*_MAX_ at low intensity contractions suggesting these values could be used interchangeably, although in the interest of rigor, it would be sensible to use a single well controlled *M*_MAX_ measure to normalize all conditions.

## Conclusion

The results from this study show that *M*_MAX_ is not altered by shortening or lengthening contraction type, but is modulated with changes in contraction intensity. Possible mechanisms may be due to the shortened muscle lengths at the higher contraction intensities. *M*_MAX_ should be used relative to task specific contraction intensities and it is vital that it is recorded under consistent reproducible conditions. No differences were seen between *M*_MAX_ at low intensity contractions and *M*_SUP_ at 100% MVC. There was also low systematic bias and strong correlations suggesting that V‐wave can be expressed relative to *M*_MAX_ recorded during low intensity contractions or *M*_SUP_ at 100% MVC. It is our recommendation that *M*_MAX_ should be recorded at specific contraction intensities but not necessarily a specific contraction type. However, consistency of *M*_MAX_ recording throughout the experiment is vital.

## Conflict of Interest

The authors have no competing interests to declare, financial, or otherwise.
